# Are urban landscapes associated with reported life satisfaction and inequalities in life satisfaction at the city level? A cross-sectional study of 66 European cities

**DOI:** 10.1016/j.socscimed.2019.03.009

**Published:** 2019-04

**Authors:** Jonathan R. Olsen, Natalie Nicholls, Richard Mitchell

**Affiliations:** MRC/CSO Social and Public Health Sciences Unit, University of Glasgow, Glasgow, UK

**Keywords:** Urban, Inequalities, Quality of life, Life satisfaction, Landscape, Ecology, Urban landscape

## Abstract

With more than half the world's population residing in urban areas and this proportion rising, it is important to understand how well-planned urban environments might improve, and reduce inequalities in, quality of life (QoL). Although studies suggest city-level characteristics hold independent influence on QoL, they generally lack a theoretically informed approach to understanding how the whole city landscape might be implicated, have paid scant attention to inequalities in QoL and often focus on small numbers of cities or countries. We applied theory and methods from landscape ecology to explore associations between cities' land cover/use, residents' reported life satisfaction and within-city socio-economic inequalities in life satisfaction. We joined individual-level responses to the European Urban Audit (EUA) Perception Surveys (2012, 2015) with city-level data from the European Urban Atlas classifying land cover/use into 26 different classes. Our sample included 63,554 people from 66 cities in 28 countries. Multilevel binary logistic models found that specific land use measures were associated with life satisfaction, including the amount of a city which was: residential (OR:0.991, 95%CI 0.984–0.997); isolated structures (OR:1.046, 95 CI 1.002–1.091); roads (OR:0.989, 95%CI 0.982–0.996); pastures (OR: 1.002, 95% CI 1.002–1.003) and herbaceous vegetation (OR:0.998, 95%CI 0.997–0.100). A more even distribution of land cover/use (β: 1.561, 95%CI -3.021 to −0.102) was associated with lower inequality in life satisfaction. This is the first study to theorise and examine how the entire urban landscape may affect levels of and inequalities in wellbeing in a large international sample. Our finding that more equal distribution of land cover/use is associated with lower levels of socio-economic inequality in life satisfaction supports the idea that city environments could be equigenic – that is, could create equality. Our findings can aid urban planners to develop and build cities that can contribute to improving, and narrowing inequalities in, residents' life satisfaction.

## Highlights

• It is important to understand how urban environments might improve quality of life.
• We explored associations between land covers and life satisfaction.
• We also explored within-city socio-economic inequalities in life satisfaction.
• Specific land use measures were associated with life satisfaction.
• Land cover evenness was associated with lower inequality in life satisfaction.

## 1 Introduction

This study is about the relationship between urban environment and residents' reported life satisfaction, an aspect of overall Quality of Life (QoL). QoL is increasingly seen as an important component of population health and wellbeing, and one which governments and policy makers are trying to maximise and equalise (Cao, 2016; Tay et al., 2015). With more than half (54%) of the world's population now residing in urban areas, and this expected to rise to two thirds (66%) by 2050 (United Nations, 2014), the influence of urban environments on all facets of health and wellbeing is particularly important to understand. In this paper we made a novel application of ideas and techniques from ecology to assess the association between entire cities' land cover (what is physically ‘on the ground’) and land use (what that ground is used for), and both life satisfaction and inequalities in life satisfaction, in a large international sample of cities.

### 1.1 Health related QoL and urban environments

There is a large and diverse literature on QoL, from many different disciplinary perspectives (Ballas and Dorling, 2013; Huynh and Peiser, 2015; Lee and Sener, 2016). We note that many disciplines have debated the underlying concepts, indicators of, and differences between, quality of life, life satisfaction, happiness and wellbeing (Tay et al., 2015), but agree with Glatzer that, in essence, they all speak to “*evaluations, both positive and negative, that people make of their lives*” (Glatzer, 2015, p. 3). One relatively consistent finding across this literature is that determinants of QoL lie at many different levels; from individual and household, through neighbourhood and city, to region and nation (Ballas and Tranmer, 2011; Węziak-Białowolska, 2016). A second finding is that, generally, inequalities in QoL are ever-present; those in less advantaged circumstances have lower QoL than those in more advantaged circumstances (Bartley, 2016).

Most studies agree that individual-level characteristics such as income and domestic life are more potent influences on QoL than environmental characteristics (Sallis et al., 2009). However, thinking about urban the environment as a lever to improve and equalise QoL remains important because, arguably, i) environmental changes can affect large numbers of people and may be easier, cheaper and more politically acceptable than intervening in the lives of individuals (Academy of Medical Sciences, 2016), and ii) urban social and physical environments change continually anyway.

Reviews of existing literature about how urban environments affect QoL note a useful divide between ‘objective’ and ‘subjective’ approaches to measuring QoL and its determinants (Ballas and Dorling, 2013; Marans and Stimson, 2011). ‘Objective’ approaches are based on assessment of social and physical environmental factors which are theorised to either reflect or determine QoL (Apparicio et al., 2008). In contrast, ‘subjective’ studies have focused on individuals' reports of their QoL, sometimes using straightforward unidimensional measures of QoL, such as a score on a life satisfaction or happiness scale, and sometimes combining measures of different facets of life together into indices (Douglas et al., 2018; Leslie et al., 2010). How humans *perceive* their local environment is important for its relationship to QoL, perhaps even more so than objective measures. For example, positive perceptions of green and open space have been found to be important predictors of greater neighbourhood satisfaction (Douglas et al., 2018). Whilst some studies have begun to compare or integrate objective and subjective approaches, this remains a surprisingly under-explored area and one in which results are mixed; often the objective and subjective assessments of QoL do not agree (Ballas and Tranmer, 2011). A substantial proportion of the literature about QoL, especially that in the ‘objective’ mould, has been dedicated to analysing and ranking QoL at city level. ‘The city’ has sometimes just been used as a useful shorthand for identifying populations of residents to compare, but the city *itself* – its characteristics, facilities, systems and form has also been identified as a significant, independent, determinant of QoL (Arundel and Ronald, 2017; Barton, 2009; Lovejoy et al., 2010).

In terms of understanding inequalities in QoL, plenty of studies examine the impact of income or socio-economic inequality *on* QoL (Schneider, 2012), but few which examine how QoL varies by income or socio-economic situation *within or between* cities. The possibility that environment itself might be able to constrain or reduce socio-economic inequalities in health has been highlighted by Mitchell et al. (2015) who suggest that a focus on ‘equigenic’ environments (those that, literally, create equality) could be fruitful (Mitchell et al., 2015).

### 1.2 Urban morphology and QoL

Urban morphology (the size, shape and configuration of the city), is one city-level characteristic which has received increasing attention for its influence on QoL (Brown et al., 2016). This focus is partly explained by the fact that many cities and city regions have planning strategies in place to control how cities grow or densify and what kinds of land may or may not be developed. Some of these plans recognise an influence on QoL (Brown et al., 2016; OECD, 2014). In other settings, planners and citizens try to keep track of, and understand the consequences of, unplanned and unregulated urban development. Within the morphology focus, urban land cover and use have both recently been included in investigations of urban QoL. Much of this work has been focused on settlement density, and on the presence of natural or open spaces within cities. In general, more open or natural space is positively associated with residents' reported QoL (Jensen and Binford, 2004; Zhang, Van den Berg, Van Dijk and Weitkamp, 2017), (though not always – see (Brown et al., 2016)) and removal or replacement of green space for urban development can impact negatively on QoL (Hrehorowicz-Gaber, 2013). However, the focus is not exclusively on natural land cover; Brown et al., (2016) also assessed relationships between the fragmentation of land use close to residents’ homes and their reported life satisfaction for example. They did so for 33 cities across 5 countries, finding that greater levels of land cover/use fragmentation around households' residence were associated with lower reported life satisfaction.

The strong foci on green space and settlement density is a weakness in the literature. Urban environments are complex, with a huge range in types of space and function; they are not simply either green or grey, or more or less densely settled. The metrics capturing land cover or land cover/use also tend to be basic, with simple quantity, proximity or proportion assessed (Krekel et al., 2016). Further, where measurements of land cover/use have been used, they have often referred to specific neighbourhoods within a city or the area around sampled residents' homes, rather than the *entire* city. This is despite a literature which, as we note above, recognises that the city itself has a role to play in affecting QoL. When we also consider research using GPS to track people's movements around their cities that shows many people spend significant time outside the areas around their homes (Chambers et al., 2017; Olsen et al., 2019), the exclusive focus on neighbourhood of residence seems inadequate (Patterson and Farber, 2015).

### 1.3 Landscape ecology and health research

Following many others within social and medical science, we identified ecology as a useful field to draw on here and landscape ecology in particular (Lovejoy et al., 2010; Pickett et al., 2016). Much of the application of ecological ideas to population health has focused on direct relationships between the natural world and the human world, and their implications for the sustainability of both (Mouratidis, 2017). However, we wish to borrow from ecology at a higher level of abstraction; we see a clear parallel between how landscape ecologists think about landscape as habitat, and how we might think about the urban environment.

Landscape ecologists recognise that organisms and their habitats exist in a complex (eco)system in which the presence, size, shape, spatial arrangement of, and balance between, different kinds of land cover affects organisms' health-related outcomes. They have developed techniques and metrics for comparing aspects of landscape (Huang et al., 2007; Wei et al., 2017). Gibson's theory of affordances also provides a framework for exploring how and *why* different land cover/uses would affect organisms (Gibson, 1979). Affordances are the possibilities the environment offers to an organism; what the environment enables it to do, for example feeding, running, drinking, climbing etc. (Jones, 2003). Where these ideas have reached towards a discussion of how affordance affects human health and wellbeing, they have largely focused on natural environments (Ward Thompson, 2011). However, we propose that together, techniques from landscape ecology and affordance theory can be used to establish new enquiry into how the mosaic of different land cover/uses that comprise an entire city might affect residents' health and wellbeing, and particularly for this study, their QoL. Although we do not intend here to contribute to the debate around ‘affordances’ theory (a more fine grained understanding of the theory exists (Lennon et al., 2017, 2019) we draw on the theory to hypothesise that the range and distribution of land covers/uses speaks to the range of opportunities and environments, and hence affordances, the city provides.

We believe there is large within-person, and between-person, variation in what is wanted or needed from the environment, moment to moment, day to day, lifestage to lifestage. Residents might not need, or want, particular things from their city all the time, or indeed often at all, but if the city can afford those things when needed, it is likely to support a higher quality of life (Douglas et al., 2017; Lennon et al., 2017; Roe et al., 2017). Specific land covers/uses might offer specific affordances. Transport infrastructure, for example, may enable people to move around to work, socialise and have leisure time. Green spaces may offer restoration from stress (Ward Thompson et al., 2012), opportunities for physical activity or social interaction (Hartig et al., 2014). This framework also enables us to consider why a city's land cover/use might affect *inequalities* in QoL. If some resources (such as green space) are very limited or spatially constrained, there might be competition to access them via the housing market, or it may be necessary to expend resources (for example to travel out of the city). Generally, more advantaged people will benefit in this situation. If adverse aspects of the environment are either very concentrated or perhaps very widespread, that too might affect inequalities as people living nearer to these may report lower QoL. Therefore, hypothesising that greater diversity of land covers/uses within a city may provide the range of ‘habitats’ that residents require for higher QoL via equality in encountering specific land-uses, both desirable and undesirable and regardless of socio-economic position, that specific land covers/uses within the city might be associated with greater or lower QoL, and that more equal distributions of different land cover/uses (as opposed to a dominance by one or two land cover/uses) might be associated with greater equality in QoL, we set and addressed our study aims.

### 1.4 Study aims

Our study aims, designed to test these ideas, were:

1) To objectively and quantitatively describe land cover/use for a large number of European cities using landscape metrics;
2) To explore the association between the availability and structure of city landscape, and residents' reported life satisfaction;
3) To explore whether the city landscape, and its aspects, are associated with socio-economic inequality in life satisfaction within cities.

## 2 Methods

### 2.1 Study areas

**Fig. 1:**
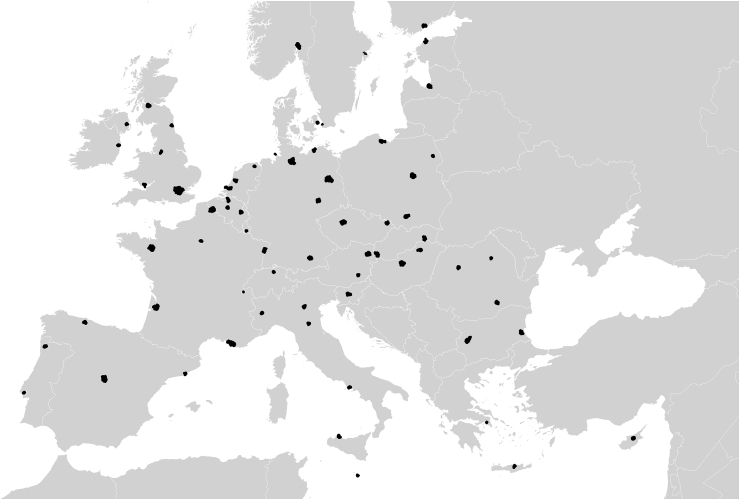
Study cities.

The study combined objective land cover/use data (the European Urban Atlas), with individual-level self-reported data about life satisfaction and other personal characteristics (the European Urban Audit Perception survey). Details of both data sets are provided below. The countries and cities used in the study are shown in Fig. 1. The final sample included 66 cities within 28 countries.

### 2.2 Data sets

#### 2.2.1 European Urban Atlas

The 2012 Urban Atlas provides pan-European comparable land cover/use data for Urban Areas and is an initiative of the European Commission with the support of the European Space Agency and the European Environment Agency (Copernicus, 2018). The Atlas has a 10 m^2^ resolution and categorises land into 26 classes (European Commission, 2012). There are four high level classes: ‘artificial surfaces’, ‘agriculture, semi-natural areas and wetlands’, ‘forests’ and ‘water’ and these are then sub-classified for greater specificity. Full land cover/use classifications and descriptions are within Supplementary Table 1. Since the Urban Audit Perception survey participants were selected if they resided within the city administrative boundary (European Commission, 2012), we ‘clipped’ the Urban Atlas geographical data to the city boundaries used in for the Urban Audit survey, within Geographical Information Systems (GIS; ArcMap 10.3 [ESRI, California]). City names have not been standaised to English and use their designations in the European Urban Atlas.

#### 2.2.2 European Urban Audit Perception survey

QoL and socio-demographic data were taken from the European Urban Audit Perception survey, years 2012 and 2015 (Directorate-General for Communication (European Commission), Directorate-General for Regional and Urban Policy (European Commission), & TNS Political and Social, 2016; Directorate-General for Regional and Urban Policy (European Commission) & TNS Political and Social, 2013). In each year, approximately 41,000 people living within the administrative boundaries of the European cities were surveyed by telephone on a range of topics that are perceived to affect urban quality of life. We pooled the data from the two survey waves to increase power, but as the survey is cross-sectional in nature, and not designed to be directly comparable across years, we could not undertake longitudinal analysis. The outcome measure came from the question “On the whole, are you very satisfied, fairly satisfied, not very satisfied or not at all satisfied with the life you lead?“. We subsequently refer to this outcome as ‘*Life Satisfaction*’. Preliminary analyses showed that the assumption of proportionality of odds required for ordinal logistic regression was violated. Responses to the question were therefore dichotomised into ‘satisfied’ (1, very satisfied and fairly satisfied) or ‘not satisfied’ (0, not very satisfied or not at all satisfied). Dichotomisation also offered the advantage of easily interpreted model output and has been previously undertaken in analysing data from Urban Audit Perception surveys (Węziak-Białowolska, 2016).

The socio-demographic variables from the surveys included: sex, age, age left education, reported difficulty paying bills at the end of the month and domestic situation. Domestic situation was available from the perception survey, and referred to the living situation of the respondent, for example with or without partner and/or children (Table 1). These were selected as well-established correlates of life satisfaction (Bowling, 1995). Difficulty in paying bills was used as a proxy for economic stability or deprivation, this is uni-dimensional indicator of economic and social circumstances which probably more strongly represents the economic, with those in the first category (Most of the time) treated as most disadvantaged. Non-responses (including “Don't Know” and “Refused”) were treated as missing values and excluded with a loss of 4% of the total number of available observations. Given this low proportion, missing data were not imputed. The survey data were then joined to the European Urban Atlas measures using city as the key. Survey weights which correct for representation were provided by the EU and applied in all models: the data were structured in a ‘long’ format with a weight provided to each individual response, as we controlled for year and weighting was applied to individual responses, thus avoiding potential double weighting. After weighting, the total sample was 63,554 individuals across the 66 cities (Table 1).

**Table 1:** Participant characteristics.

Variable	Number	Proportion	% satisfied
**Gender**			
Male	30019	47.2	87.8
Female	33535	52.8	86.8
**Age**			
15–24 years	7425	11.7	92.1
25–39 years	20172	31.7	89.0
40–54 years	14834	23.3	85.1
55 years +	21123	33.2	85.5
**Difficulty paying bills**			
Most of the time	6984	11.0	59.8
From time to time	13355	21.0	82.9
(Almost) Never	43216	68.0	93.1
**Age left education**			
None or to less than 16	5570	8.8	79.9
16–19 years	19728	31.0	83.8
20 years or more	32860	51.7	89.4
Still studying	5396	8.5	94.5
**Domestic situation**			
Single no children	13693	21.5	85.7
Single, 1 + children	4947	7.8	81.2
Married/cohabiting no children	16170	25.4	89.8
Married/cohabiting, 1 + children	23756	37.4	88.4
Other	4989	7.8	84.1

**Overall**	63554	100	87.3
**Median city life satisfaction value (IQR)**			89.6 (84.2–93.4)
**Range by city**			52.9 to 97.5

### 2.3 Analysis

#### 2.3.1 Describing urban landscapes

We calculated metrics to describe each city's landscape using an extension for ArcMap 10.3 GIS called Patch Analyst (R. S. Rempel, Kaukinen and Carr, 2012). The process works by identifying ‘patches’ of land cover/use within the landscape under analysis. A patch is a singular uninterrupted polygon area of land of any size or boundary shape. The number, size, shape and relative location of patches of each land cover/use then form the basis of a variety of possible metrics for the landscape (in this case, a city) and metrics produced for each city are comparable.

##### 2.3.1.1 City land cover/use and cover descriptions: diversity and evenness

We used two metrics to define the diversity and evenness of land cover/use within a city; Shannon's diversity index (SDI) and Shannon's Evenness index (SEI).

###### 2.3.1.1.1 City land cover/use diversity (SDI)

**Fig. 2:**
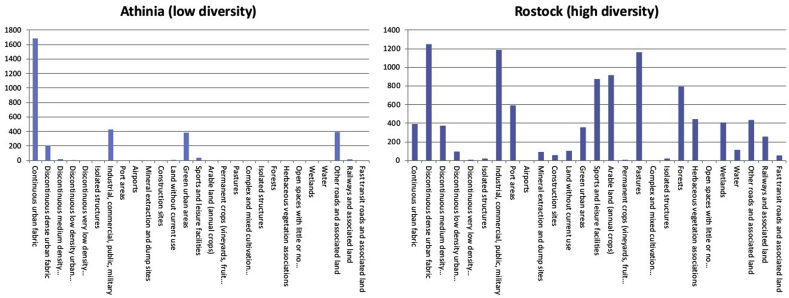
Least and most diverse European cities calculated using Shannon's diversity index (SDI).

Land cover/use diversity within the administrative boundary was measured for each city using the SDI. The SDI is interpreted as: “*a measure of relative patch diversity available at the landscape level and is a relative measure of patch diversity. The index will equal zero when there is only one patch in the landscape and increases as the number of patch types or proportional distribution of patch types increases.*” (R. Rempel, Carr and Elkie, 2008). Fig. 2 helps to illustrate what the diversity metric captures by showing the distribution of land cover/uses across the two cities in our sample with the lowest and highest SDI index values. Athinia is dominated by one land cover/use (continuous urban fabric). Rostock however, contains a wide range of different land covers/uses within its city boundary.

###### 2.3.1.1.2 City land cover/use evenness (SEI)

**Fig. 3:**
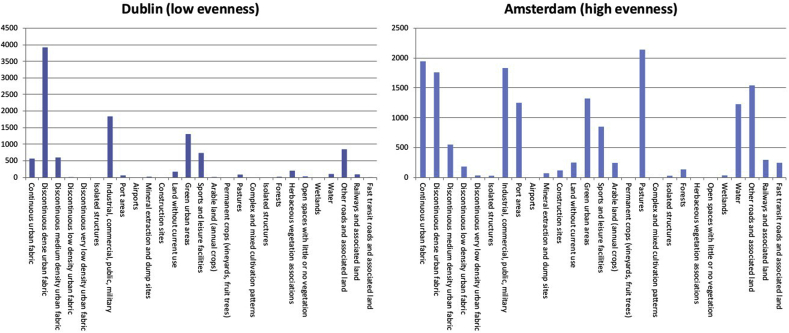
Least and most even European cities calculated using Shannon's Evenness index (SEI).

To quantify the evenness of land cover/uses within each city, we computed the Shannon's Evenness index (SEI). This is interpreted as: “*a measure of patch distribution and abundance. SEI is equal to zero when the observed patch distribution is low and approaches one when the distribution of patch types becomes more even.*” (R. Rempel et al., 2008). Fig. 3 helps to illustrate what SEI measures by comparing examples of a city with lower SEI score (Dublin) and a higher SEI score (Amsterdam). Dublin contains multiple land-uses but there is considerable variation in the relative total sizes of these landscape components. In contrast, Amsterdam had many land covers/uses with more similar total areas.

###### 2.3.1.1.3 Individual land cover/use metrics

We then calculated the total amount of each city's land area classified into each of the 26 land cover/use classifications provided by the Urban Atlas. We also calculated the total land area for each city, which was used as a control in the models, effectively rendering our metrics as a proportion. Finally, we computed the total ‘residential’ component of the city by summing the values for all artificial surfaces that were predominately classified as residential.

### 2.4 Statistical analysis

#### 2.4.1 Exploring associations between the availability and structure of city landscape, and residents’ reported life satisfaction

Descriptive statistics in terms of percentages in each category, reporting life satisfaction or not, were generated for each socio-demographic variable (gender, age, age left education, difficulty paying bills and domestic situation). The life satisfaction measure was then treated as the outcome for a series of mixed binary logistic models which allowed for the clustered nature of the data (individual, within city, within country). In all models, year of survey, the socio-demographic variables and land cover/use measures were all fitted as fixed effects, and city, nested within country, was fitted as a random effect. Separate models were run for each land cover/use metric. Total city land area was also controlled for in all models apart from those for diversity, evenness and the proportion of city area that was residential.

#### 2.4.2 Exploring whether urban landscape is associated with socio-economic inequality in life satisfaction within cities

To capture the socio-economic inequality in reported life satisfaction, a version of the slope index of inequality (Pamuk, 1985) was calculated for each city. A logistic regression model was performed for each city with life satisfaction as the dependent variable and year of survey, sex, age, age left education, domestic situation and difficulty paying bills as independent variables. In contrast to the previous analysis, difficulty paying bills was treated as a continuous variable in these models to obtain a single coefficient representing the gradient of inequality of satisfaction across the three groups: the difference in satisfaction between the most economically stable and least, while controlling for other factors. The coefficient for difficulty paying bills, in effect the slope of its association with life satisfaction, was extracted as a summary of the socio-economic inequality in life satisfaction for each city. All values were positive, indicating an increased likelihood of satisfaction with increasing socio-economic advantage. Variation in the size of the coefficient indicated the ‘steepness’ of socio-economic gradient in life satisfaction for that city. This coefficient was then used as the dependent variable in linear regressions then performed separately for each land cover/use measure, controlling for country and total city land area.

All analyses were performed in Stata (SE) v14 (StataCorp, 2015), with significance level set at 0.05.

## 3 Results

### 3.1 Description of urban land cover/use across European cities

**Fig. 4:**
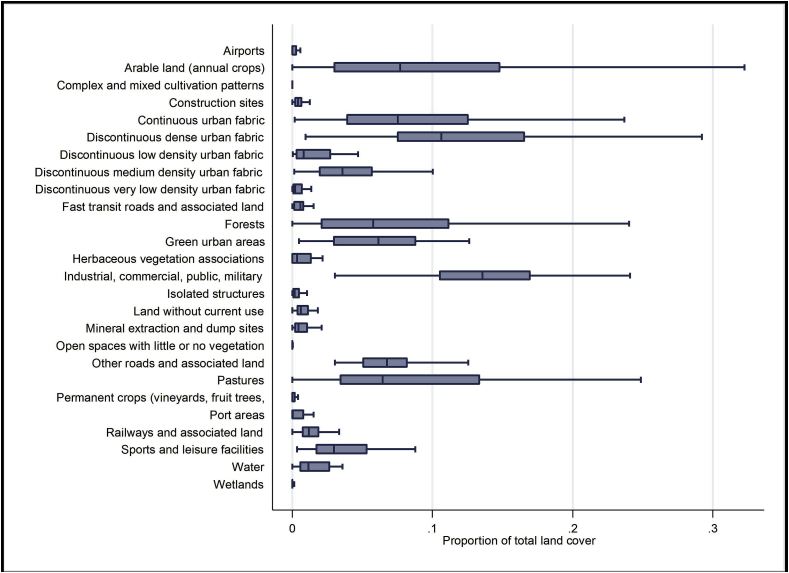
Box plot representing summary proportion of total land. Note: Rectangular box represents the second and third quartiles, the line within the box is the median value. The whiskers show the range of values.

Fig. 4 shows the median proportions of each land cover/use class. The dominant land cover/use characteristic for all cities were residential areas (continuous and discontinuous urban fabric), industrial, commercial and public lands, and natural land types (arable land, forests, green urban areas, and pastures).

**Fig. 5:**
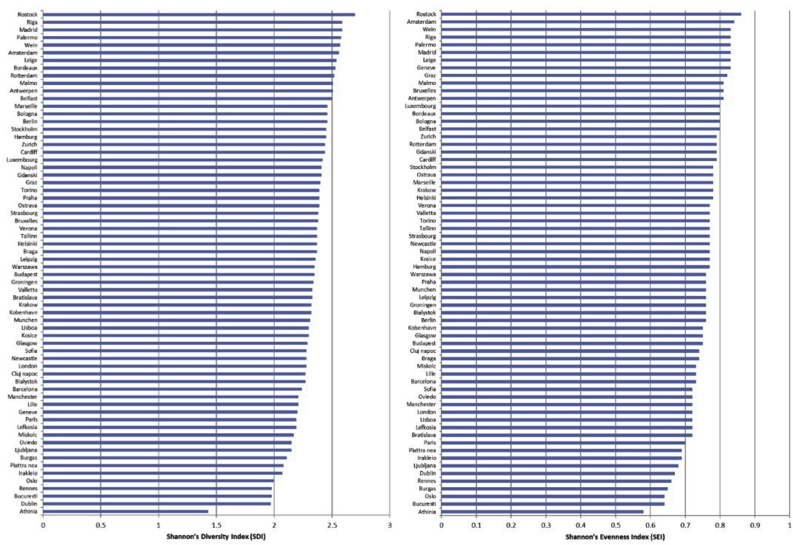
Shannon’s diversity index (SDI) and Shannon’s evenness index (SEI) by city (higher number indicates greater diversity and evenness of land-uses within a city).

Fig. 5 shows the SEI and SDI figures for each city, ranked by scores and shows that cities demonstrate measurable differences in relation to these metrics. There was substantial variation in both measures, illustrating the variety of urban forms within the sampled cities. These two measures were highly correlated (Spearman's Rank = 0.88, p < 0.001).

### 3.2 Life satisfaction

Overall, the median value for life satisfaction was 89.6% (Range (by city) 52.9%–97.5%), there was little difference in this figure between sexes (males 87.8%, females 86.8%). Younger individuals (92.1%), those almost never having difficulty paying bills (93.1%), those staying in education longer (89.4%) or still studying (94.5%), and those being married without children (89.8%), had greater life satisfaction (Table 1).

**Fig. 6:**
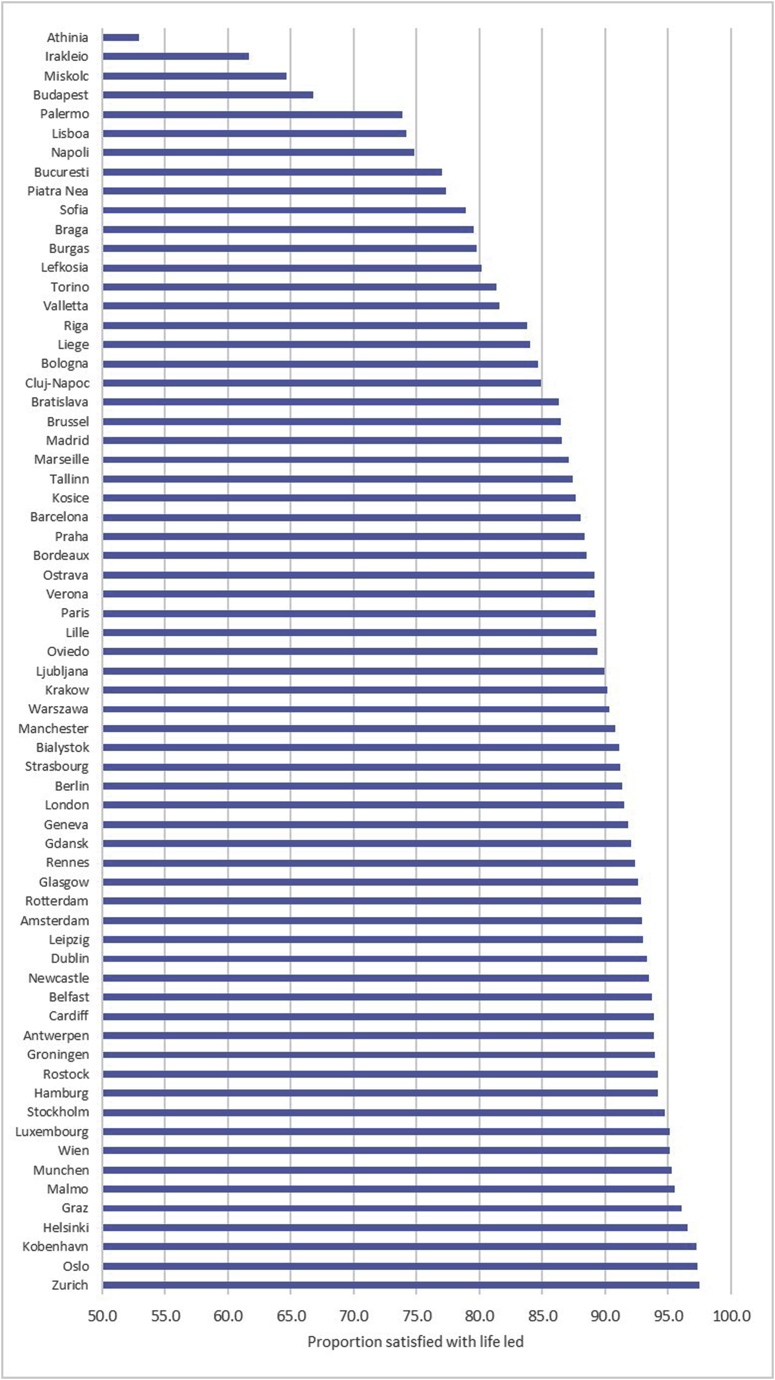
Life satisfaction by city.

Life satisfaction varied between individual cities: the cities where individuals reported the greatest satisfaction were Zurich (97.5%), Oslo (97.3%) and Kobenhavn (97.3%) (Fig. 6). The Greek and Hungarian cities of Athina (52.9%), Irakleio (61.7%) and Miskolc (64.7%) reported the lowest life satisfaction.

### 3.3 Associations between availability and structure of city landscape, and residents’ reported QoL

The metrics SDI and SEI were not associated with life satisfaction (Table 2). The odds ratios (OR) for the land covers/uses in Table 2 can be interpreted as the likelihood of satisfaction with life per additional square kilometre of land cover/use, after accounting for the other confounding variables. The amount of some land covers/uses present in the city was associated with higher life satisfaction: arable land (Odds ratio (OR) 1.002, 95% CI 1.000 to 1.003), pastures (OR 1.002, 95% CI 1.002 to 1.003), and isolated structures (residential dwellings surrounded by natural green space (OR 1.046, 95% CI 1.002 to 1.091)). Land covers/uses associated with lower life satisfaction were continuous urban fabric (OR 0.998, 95% CI 0.997 to 0.999), industrial, commercial, public and military (OR 0.994, 95% CI 0.991 to 0.998), other roads and associated land (OR 0.989, 95% CI 0.982 to 0.996), green urban areas (OR 0.993, 95% CI 0.988 to 0.997), herbaceous vegetation (OR 0.998, 95% CI 0.997 to 0.999) and complex cultivation patterns (OR 0.861, 95% CI 0.806 to 0.920). Table 2 also provides the estimated ORs for two more meaningful measures – the 25th and 75th percentile of the land cover/use.

**Table 2:** Life satisfaction by amount of land cover/use characteristic within cities (weighted).

Land cover/use	OR	LL 95% CI	UL 95% CI	Est. 25th percentile OR	Est.75th percentile OR	P value
Residential proportion*	**0.991 **	**0.984 **	**0.997 **	**0.802**	**0.654**	**0.006 **
Continuous urban fabric*	**0.998 **	**0.997 **	**0.9995**	**0.990**	**0.954**	**0.008 **
Discontinuous dense urban fabric	0.999	0.997	1.001	0.992	0.966	0.334
Discontinuous medium density urban fabric	0.999	0.996	1.002	0.998	0.987	0.603
Discontinuous low density urban fabric	1.000	0.983	1.017	1.000	1.000	0.999
Discontinuous very low density urban fabric	1.032	0.997	1.068	1.005	1.035	0.073
Isolated structures*	**1.046 **	**1.002 **	**1.091 **	**1.006**	**1.049**	**0.039 **
Industrial, commercial, public, military*	**0.995 **	**0.991 **	**0.998 **	**0.936**	**0.792**	**0.005 **
Port areas	1.008	0.998	1.018	1.000	1.010	0.112
Airports	0.984	0.946	1.024	1.000	0.991	0.437
Other roads and associated land*	**0.989 **	**0.982 **	**0.996 **	**0.920**	**0.830**	**0.003 **
Railways and associated land	1.013	0.963	1.067	1.011	1.056	0.614
Fast transit roads and associated land	1.005	0.956	1.057	1.001	1.011	0.849
Open spaces with little or no vegetation	0.902	0.730	1.115	1.000	0.997	0.342
Mineral extraction and dump sites	0.995	0.979	1.012	0.998	0.990	0.564
Construction sites	1.006	0.971	1.043	1.002	1.008	0.729
Land without current use	0.989	0.975	1.003	0.995	0.978	0.136
Green urban areas*	**0.993 **	**0.988 **	**0.997 **	**0.972**	**0.879**	**0.002 **
Sports and leisure facilities	0.993	0.984	1.002	0.984	0.937	0.107
Arable land (annual crops)*	1.002	1.000	1.003	1.008	1.061	0.028
Permanent crops (vineyards, fruit trees etc.)	1.006	0.986	1.026	1.000	1.002	0.545
Pastures*	**1.002 **	**1.002 **	**1.003 **	**1.008**	**1.049**	<**0.001 **
Complex and mixed cultivation patterns*	**0.861 **	**0.806 **	**0.920 **	**1.000**	**1.000**	<**0.001 **
Herbaceous vegetation associations^1^	0.998	0.997	1.000	1.000	0.996	0.028
Wetlands	0.983	0.959	1.007	1.000	0.997	0.154
Forests	1.000	0.996	1.003	1.000	1.000	0.777
Water	1.004	0.990	1.019	1.003	1.028	0.555
						
SDI	1.425	0.777	2.611	1.950	2.039	0.252
SEI	2.714	0.347	21.232	2.241	2.359	0.342
						
Null model variance (ICC): Country	0.153	0.101	0.224			
Null model variance (ICC): City in Country	0.167	0.116	0.235			

*p < 0.05.

Models performed separately for individual land-uses. Adjusted for: Age, Sex, Age left education, domestic situation and difficultly paying the bills.

The estimated percentile ORs are derived by multiplying the differences from 1 (of each model OR) by metric value at the 25th and 75th percentile, then reading 1.

1 Not adjusted for Age left education due to non-convergence.

### 3.4 Socio-economic inequality in life satisfaction within cities

**Fig. 7:**
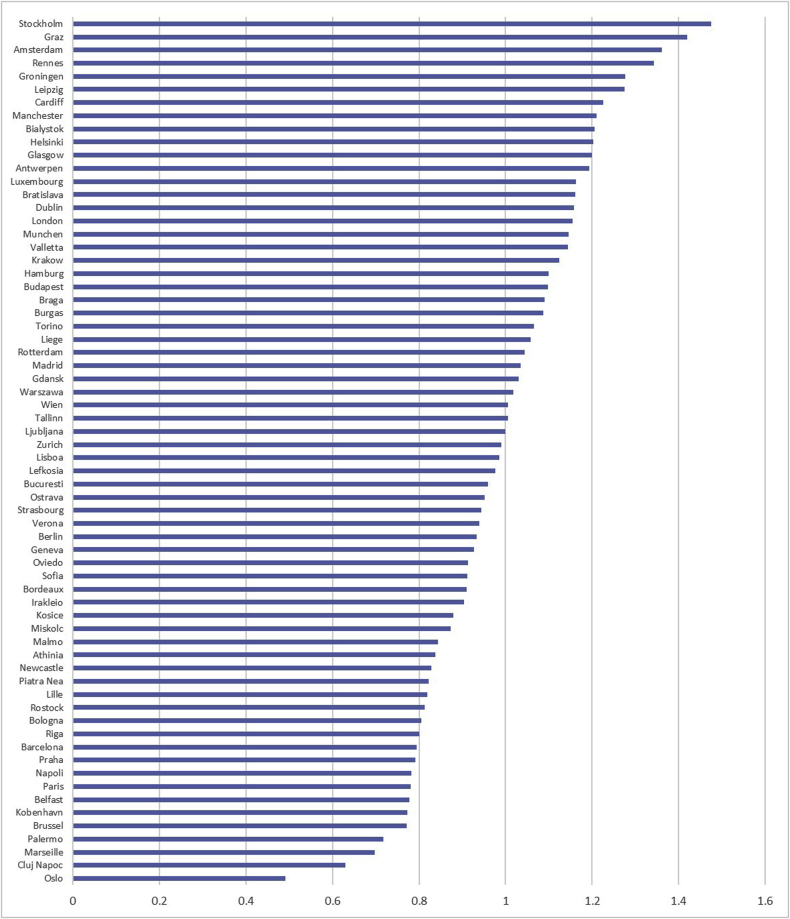
Socio-economic inequality coefficient by city.

Fig. 7 presents the inequality coefficient for each city; a higher coefficient represents a greater level of socio-economic inequality in life satisfaction within a city. Stockholm, a city with overall high levels of life satisfaction, has the largest coefficient, showing that, in comparison to the other 65 cities, there is greater inequality in life satisfaction between individuals. Oslo has the lowest coefficient, showing this city has the least inequality in life satisfaction compared to the other cities.

The inequality coefficient was associated with the amount of some land cover/uses within a city and the evenness of those land cover/uses across the city landscape (Table 3). Increased proportions of low density urban fabric and of pasture were associated with increased inequality within a city, and the association for isolated structures (i.e. singular dwelling structures surrounded by green space) was very close to significance. More even distribution of land cover/uses within a city was associated with lower levels of socio-economic inequality in life satisfaction (β: 1.561, 95% CI -3.021 to −0.102).

**Table 3:** Associations between Inequality coefficient and land cover/use within cities (weighted).

Land cover/use	Coefficient	LL 95% CI	UL 95% CI	P value
Residential proportion	−0.001	−0.007	0.005	0.852
Continuous urban fabric	−0.001	−0.003	0.001	0.416
Discontinuous dense urban fabric	−0.001	−0.004	0.002	0.361
Discontinuous medium density urban fabric	−0.001	−0.005	0.004	0.797
Discontinuous low density urban fabric	0.004	−0.012	0.020	0.595
*Discontinuous very low density urban fabric*	**0.056**	**0.007**	**0.106**	**0.027**
*Isolated structures*	*0.030*	*0.000*	*0.061*	*0.051*
Industrial, commercial, public, military	−0.001	−0.007	0.004	0.658
Port areas	−0.001	−0.008	0.005	0.695
Airports	0.000	−0.044	0.044	0.986
Other roads and associated land	−0.006	−0.017	0.005	0.298
Railways and associated land	−0.020	−0.050	0.009	0.171
Fast transit roads and associated land	−0.009	−0.051	0.033	0.669
Open spaces with little or no vegetation	0.207	−0.463	0.877	0.535
Mineral extraction and dump sites	−0.004	−0.036	0.029	0.814
Construction sites	0.009	−0.051	0.069	0.753
Land without current use	0.004	−0.022	0.030	0.766
Green urban areas	−0.004	−0.011	0.004	0.300
Sports and leisure facilities	−0.008	−0.020	0.003	0.160
Arable land (annual crops)	0.001	−0.001	0.003	0.204
Permanent crops (vineyards, fruit trees etc.)	−0.002	−0.015	0.010	0.692
Pastures	**0.002**	**0.000**	**0.004**	**0.048**
Complex and mixed cultivation patterns	0.139	−0.171	0.449	0.370
Herbaceous vegetation associations	−0.001	−0.004	0.002	0.563
Wetlands	−0.016	−0.087	0.055	0.650
Forests	−0.001	−0.005	0.002	0.427
Water	0.005	−0.007	0.017	0.407
SDI	−0.340	−0.733	0.053	0.088
*SEI*	**−1.561**	**−3.021**	**−0.102**	**0.037**

Bold coefficients are significant at p < 0.05, italicised coefficients are within 0.01 of significance.

## 4 Discussion

The aims of this study were: to describe urban landscape for a large number of European cities; explore its association with residents' reported life satisfaction; and explore whether the urban landscape was correlated with socio-economic inequality in life satisfaction within cities. We hypothesised that greater diversity of land covers/uses within a city may provide the range of ‘habitats’ that residents require for higher life satisfaction, that specific land covers/uses within the city might be associated with greater or lower life satisfaction, and that more equal distributions of different land cover/uses (as opposed to a dominance by one or two land cover/uses) might be associated with greater equality in life satisfaction.

Our hypotheses were partially supported. We did *not* find variety or distribution of land covers/uses in a city to be associated with reported life satisfaction, but we *did* identify specific land cover/uses whose areas were associated with life satisfaction. We also found that specific land cover/uses, and the evenness of the land cover/use distribution, were both positively associated with socio-economic inequalities in life satisfaction within the city. This large, international study of 66 cities suggests it is useful to consider the entire urban landscape as a contributor to both life satisfaction and inequalities in life satisfaction within the city. It is the first study to consider how urban landscape affects inequalities in life satisfaction, and not just life satisfaction itself.

Several specific land covers/uses associated with life satisfaction were closely linked to the presence or absence, and the type, of nature or green space. Having open farmland (arable and pasture) within the city boundary was positively, though weakly, associated with life satisfaction, whereas herbaceous vegetation (essentially scrub land) and green urban areas were negatively associated. The positive association of life satisfaction with ‘isolated structures’ (very low-density settlement in which residences are surrounded by natural spaces) was one of the strongest relationships. Numerous studies have found a positive relationship between the presence of green/natural spaces and life satisfaction scores (Krekel et al., 2016; Ma et al., 2018; Zhang et al., 2017). A German study for example found a negative association between increased distance between open spaces and home, and life satisfaction (Krekel et al., 2016). The positive association between ‘natural’ farmland and life satisfaction is therefore, as expected, but our observed negative association with other types of ‘natural’ space, particularly ‘urban green space’, and life satisfaction is at first sight, surprising.

It is important to consider the definition of urban green space within the European Urban Atlas when interpreting these findings. The Urban Atlas definition excluded sports fields, pastures, woodland and herbaceous vegetation areas; as these were defined as separate land-uses. In these data, urban green spaces were *any* other permeable, soft surfaces such as soil, grass, shrubs, trees and urban parkland.

Other specific land covers/uses were negatively associated with life satisfaction and these results were largely congruent with the existing (albeit limited) literature. We observed a negative association between life satisfaction and ‘other roads’, which covers all roads apart from fast transit/motorways. Living near to roads has been shown to negatively influence life satisfaction. A study of European Union citizens, for example, found a negative association from roads via air and noise pollution, and via sleep disturbance (Urban and Máca, 2013). That study also noted that other noisy transport infrastructure, such as rail, did not seem to adversely affect life satisfaction (Urban and Máca, 2013) and this echoes our observation that rail infrastructure in the urban area was not associated with life satisfaction. We found that continuous urban fabric, (i.e. areas where housing, shops and sealed/concrete surfaces dominate), and industrial, commercial, public and military areas (defined largely by the absence of vegetation and presence of concrete/tarmac) were also associated with lower life satisfaction. Cities with large amounts of this land cover are plausibly also ‘compact cities’; an urban design and development plan that is advocated by the American Planning Association, European Commission and United Nations (Mouratidis, 2017). The definition of a compact city can vary but, in essence, they are dense and develop via ‘in-fill’ and redevelopment of existing spaces, as opposed to developing outwards with low-density urban sprawl. Although the compact city is considered more sustainable (owing to the reduced carbon emissions required in intra-urban transport and service) (Jabareen, 2006), several studies suggest this form of settlement is associated with lower liveability (Neuman, 2005), although this is disputed (Mouratidis, 2017). A study which assessed the urban structure of 20 European (within 4 countries) and 33 Japanese cities, for example, found that life satisfaction scores were lower in cities that were more compact (Brown et al., 2016). We had anticipated the possibility that industrial, commercial, public and military areas might be positively associated with QoL because they afford employment and economic opportunities. It is possible that our adjustment for socio-economic situation (closely related to employment status), removed this potential benefit.

We had hypothesised that a higher diversity of land covers/uses, and more even distribution, would be associated with greater life satisfaction in the city, but this was not the case. Given that relatively few of the land covers/uses our data captured were related individually to life satisfaction, perhaps many of the land covers/uses we could distinguish in our data did not capture very well all the things residents need from their urban environment and hence their range and distribution were also not related to life satisfaction.

We found a more even distribution of land covers/uses was associated with lower inequality in life satisfaction within the city, but also that low density urban fabric, pastures and (weakly) isolated structures were associated with higher inequalities in life satisfaction. The specific land covers/uses are usually associated with residences of individuals of high socio-economic position. We note that there were more land covers/uses associated with worse life satisfaction and that these land covers/uses also tended to be more common (i.e. to have a lager extent in the city) (Fig. 4). It is possible that the positive impact of evenness in land cover/use is actually highlighting cities in which these more common uses are counter-balanced by a greater presence of others. Although it is well established that cities which contain large spatial concentrations of poverty can have a negative effect on those living in them (Ostendorf et al., 2001) relatively little is known about the effect of a greater spatial concentration of wealth in a city upon population level life satisfaction. Our finding could be due to an increase in these affluent land covers/uses extenuating social segregation and visibility of this segregation (Musterd, 2006). Although we controlled for individual socio-economic status, the visibility and spatial segregation of the form of a city may still have an impact on life satisfaction. The finding that more even distribution of land covers/uses was associated with lower inequalities suggests that this facet of urban landscape might be ‘equigenic’ – that is, promoting equality. Where the city hosts different land covers/uses (and, according to our hypothesis, hence affordances) in a balanced, even manner, perhaps access to affordances is also equalised.

### 4.1 Study strengths and limitations

This was a large international study, including 66 European cities across 28 counties. We drew on high quality survey data; they were gathered on a consistent basis, using an appropriate sampling frame and with low levels of missing values. By using the European Urban Atlas to objectively measure urban landscapes, we were able to compare cities on a consistent basis. The inclusion of so many countries and cities makes our results likely generalisable to many other developed world settings (although those in countries with very different and distinct urban forms, such as the USA, may need to be more cautious). The inclusion of multiple cities for most of the 28 countries and use of mixed models, meant we were able to allow for country-level influences on life satisfaction (including country-specific cultural reporting bias). Whilst other studies have looked at associations between aspects of urban form and life satisfaction, our study is unique in terms of the number of land cover/use types considered and the application of landscape metrics to look at evenness and diversity in land cover/use. This is also the first study to examine the association between urban landscape and inequalities in life satisfaction within a city. We had a clear theoretical framework underpinning our hypotheses.

Although we included two waves of the repeat-cross sectional European Urban Audit Perception study, we only measured land cover/use at one point in time rendering the study cross-sectional and therefore unable to determine causality. We chose to use two metrics to describe the evenness and diversity of the land structures within each city. Other metrics are available and may have given different results. However, the SDI and SEI are two commonly used measures and have previously been used in other studies to describe the distribution of land cover (Li et al., 2011; Wu et al., 2014). These metrics describe each city as a whole but did not capture the spatial distribution of the land cover/uses *within* the city, meaning that they are measures of statistical, rather than spatial, distribution. This, combined with being unable to assess which part of a city individual respondents resided in (the data did not disclose a more precise spatial location) or where they travelled within the city, meant we had no information on how individuals were actually exposed to the land cover/uses we measured. A great deal of literature on how urban areas affect QoL examines neighbourhood level influences. Since we did not have data at the neighbourhood level, we were only able to model as individual, nested within city, nested within country. Some of the variation in life satisfaction we have ascribed to city level influence may well actually reside at different spatial scales. Further, the boundaries of the cities were administrative rather than by actual settlement. Although we made efforts to ensure cities whose administrative boundary encompassed clearly rural areas were excluded from the analysis, there may be a number of individuals included who resided in rural settings.

Our hypotheses were based on analogies between how landscapes affect organisms, and how cities might affect humans. Whilst these ideas are plausible, we do not know exactly how and why different land covers/uses would affect humans and their life satisfaction and whether the affordance hypothesis is correct. As noted already, the land cover classes were limited in the extent to which they captured what affordance each environment would provide. Further, existing theories of QoL acknowledge that how humans perceive their environment is as, if not more, important as what their environment may objectively be. Our outcome variable was life satisfaction, which is an operationalisation of QoL but there are clear concepts, indicators of, and differences between, QoL, life satisfaction, happiness and wellbeing.

## 5 Conclusions

The content and form of a whole city may influence how residents feel about their lives, and the level of socio-economic inequality in that within the city too. Cities which have a more equal balance in land seem to enjoy lower levels of socio-economic inequality in life satisfaction. These findings may aid urban planners to develop and build cities that can contribute to improving population life satisfaction and narrowing inequalities. In this study we drew usefully on ideas and methods from ecology, and landscape ecology in particular. We believe that public health and ecology can learn more from each other, aside from the (important) understanding of how human and natural systems are inter-twined in issues of sustainability. The next steps for this work should be a better understanding of how humans *use* their urban habitats at multiple and varying time scales, perhaps derived from studies using GPS trackers. With better understanding of how people of different ages, life stages and situations use (or not) their urban landscapes may come more potent interventions to improve health and wellbeing, and reduce health inequalities.

## Conflicts of interest

The authors declare that there are no conflicts of interest.

## Funding statement

JO, NN and RM are employed by the University of Glasgow and funded as part of the Neighbourhoods and Communities Programme (MC_UU_12017/10) (SPHSU10) at the MRC/CSO Social and Public Health Sciences Unit (SPHSU).

## Data sharing statement

European Urban Audit and European Urban Atlas data are freely available from Eurostat (http://ec.europa.eu/eurostat/web/cities/data/database) and European Environment Agency (https://www.eea.europa.eu/data-and-maps/data/urban-atlas), both provided by the European Commission of the European Union.
